# Impact of Silicon Nanoparticles on the Antioxidant Compounds of Tomato Fruits Stressed by Arsenic

**DOI:** 10.3390/foods8120612

**Published:** 2019-11-23

**Authors:** Magín González-Moscoso, Nadia Valentina Martínez-Villegas, Gregorio Cadenas-Pliego, Adalberto Benavides-Mendoza, María del Carmen Rivera-Cruz, Susana González-Morales, Antonio Juárez-Maldonado

**Affiliations:** 1 Doctorado en Ciencias en Agricultura Protegida, Universidad Autónoma Agraria Antonio Narro, Saltillo, Coahuila 25315, Mexico; magingonmos@gmail.com; 2 IPICyT, Instituto Potosino de Investigación Científica y Tecnológica, San Luis Potosí SLP 78216, Mexico; nadia.martinez@ipicyt.edu.mx; 3 Centro de Investigación en Química Aplicada, Saltillo, Coahuila 25294, Mexico; gregorio.cadenas@ciqa.edu.mx; 4 Departamento de Horticultura, Universidad Autónoma Agraria Antonio Narro, Saltillo, Coahuila 25315, Mexico; adalberto.benavides@uaaan.edu.mx; 5 Colegio de Postgraduados Campus Tabasco, H. Cárdenas, Tabasco 86570, Mexico; mariari@colpos.mx; 6 CONACyT-Departamento de Horticultura, Universidad Autónoma Agraria Antonio Narro, Saltillo, Coahuila 25315, Mexico; qfb_sgm@hotmail.com; 7 Departamento de Botánica, Universidad Autónoma Agraria Antonio Narro, Saltillo, Coahuila 25315, Mexico

**Keywords:** bioactive compounds, oxidative stress, lycopene, hydrogen peroxide, β-carotene

## Abstract

Tomato fruit is rich in antioxidant compounds such as lycopene and β-carotene. The beneficial effects of the bioactive compounds of tomato fruit have been documented as anticancer activities. The objective of this research was to determine whether arsenic (As) causes changes in the content of antioxidant compounds in tomato fruits and whether Silicon nanoparticles (SiO_2_ NPs) positively influence them. The effects on fruit quality and non-enzymatic antioxidant compounds were determined. The results showed that As decreased the oxide-reduction potential (ORP), while lycopene and β-carotene were increased by exposure to As at a low dose (0.2 mg L^−1^), and proteins and vitamin C decreased due to high doses of As in the interaction with SiO_2_ NPs. A dose of 250 mg L^−1^ of SiO_2_ NPs increased glutathione and hydrogen peroxide (H_2_O_2_), and phenols decreased with low doses of As and when they interacted with the NPs. As for the flavonoids, they increased with exposure to As and SiO_2_ NPs. The total antioxidant capacity, determined by the ABTS (2,2´-azino-bis[3-ethylbenzthiazolin-6-sulfonic acid]) test, showed an increase with the highest dose of As in the interaction with SiO_2_ NPs. The application of As at low doses induced a greater accumulation of bioactive compounds in tomato fruit; however, these compounds decreased in high doses as well as via interaction with SiO_2_ NPs, indicating that there was an oxidative burst.

## 1. Introduction

Tomato is the second most important crop in the world from an economic point of view [1]. However, tomato fruit is also important in the human diet because it can provide vitamins and a wide range of bioactive molecules [2], such as vitamins C and E, flavonoids, and phenols [3]. Lycopene is one of the strongest natural antioxidants known and is the main carotene in ripe tomatoes [4], and together with β-carotene, it is effective in eliminating peroxyl radicals [5]. These compounds seem to play a role against the development of different types of cancer and cardiovascular diseases due to their antioxidant capacity [6,7], which is why they are very important for human health.

There are many biotic and abiotic factors that influence the growth and development of a tomato crop, which can modify the quality of the fruit. One of these is arsenic (As), a highly toxic metalloid for plants and humans [8,9]. It is known that irrigation water contaminated by As causes a gradual and continuous accumulation in the soil that affects the sustainability of agriculture, decreases crop yield, and contaminates the food chain [10,11]. The concentration of As in rice grains has been reported to range from 93 to 989 µg kg^−1^ dry weight [12], in mango fruits from 0.6 to 50 µg kg^−1^ fresh weight [13], and from 2 to 13 µg g^−1^ of dry weight in tomatoes [14]. In addition, the capacity of a tomato plant to absorb and translocate As is of vital importance, since the fruit is the organ of consumption [15].

Arsenic induces the production of reactive oxygen species (ROS), which lead to lipid peroxidation [16,17]. Antioxidant metabolites synthesized by plants to avoid ROS-induced damage during oxidative stress are constitutively present in fruits and at different levels according to their stage of maturation [18]. The oxidative stress in tomato fruit increases in a coordinated manner with the ripening of the fruit and reaches a peak in the final stages, which triggers metabolic changes and softening of the fruit [19].

Currently, nanotechnology is used for different purposes in agriculture for the promotion of plant growth [20]. Nanoparticles (NPs, materials with a dimension of less than 100 nm) are noteworthy and can be considered biostimulants, since in specific ranges of concentration, generally at low levels, they increase plant growth [21]. NPs have been implicated in inducing a better quality of tomato fruit and increasing lycopene under conditions of abiotic stress [22]. Silicon nanoparticles (Si NPs) increase the yield of cucumber fruit [23]. Silicon decreases the toxic effect of heavy metals by reducing uptake and translocation to the aerial parts of plants [24]. It can also inhibit the negative impact of oxidative stress caused by As by restricting the production of ROS, improving the action of various antioxidant compounds, and regulating the osmotic potential of the cell [25]. Considering the above, the objective of the present investigation was to determine the impact of the SiO_2_ NPs on the antioxidant content of tomato fruits obtained from plants developed under conditions of arsenic stress.

## 2. Materials and Methods 

### 2.1. Fruit Sampling

A tomato crop (*Solanum lycopersicum* L.) var. “Sun 7705” (Nunhems Amsterdam Netherlands B.V. Napoleonsweg 152 6083 AB Nunhem Nederland), of type saladette and indeterminate, was grown. A total of 216 plants were established for the experiment. A soil-less culture system was used, which involved placing the plants in 12 L black polyethylene bags containing a mixture of Peat moss and perlite (1:1) as substrate. A Steiner nutrient solution [26] was used for crop nutrition.

Arsenic contamination was simulated in the irrigation water, where different concentrations of the contaminant were applied (0, 0.2, 0.4, 0.8, 1.6, and 3.2 mg As L^−1^ water). In addition, different doses of silicon dioxide nanoparticles (SiO_2_ NPs) (0, 250, and 1000 mg L^−1^) were applied. The SiO_2_ NPs (10 mL to each plant) were applied to soil at intervals of three weeks from transplanting, with a total of six applications. In total, 18 treatments were evaluated. SiO_2_ NPs of 10–20 nm size had a spherical morphology, a surface area of 160 m^2^ g^−1^, and a bulk density of 0.08–0.1 g cm^−3^ (SkySpring Nanomaterials Inc., Houston, TX, USA). Arsenic was applied as sodium arsenate heptahydrate (Na_2_HAsO_4_·7H_2_O) in the irrigation water.

It was verified that the tomatoes selected by treatment did not have damage and were of a homogeneous size. They were harvested as full red according to the USDA scale: red indicates that more than 90 percent of the surface in the aggregate shows a red color [27]. Quality analysis, biochemical analysis, and an arsenic content determination were performed on these fruits.

### 2.2. Fruit Quality

The parameters that describe a fruit’s quality (hydrogen potential (pH), total soluble solids (TSS), fruit firmness, titratable acidity (TA), electrical conductivity (EC), and Oxide-Reduction Potential (ORP) were determined, as described in López-Vargas et al. [28]. For this, six fruits per treatment (one per plant) of uniform size and in a light red state of maturity were collected from the third cluster.

### 2.3. Biochemical Analysis

The fruit samples were stored at −80 °C until use. The samples were lyophilized (lyophilizer, Yamato Scientific Co. Ltd., Model D401, Santa Clara, CA, USA). For antioxidant compound determination, 200 mg of lyophilized fruits and 20 mg of polyvinylpyrrolidone were weighed. After this, 1.5 mL of phosphate buffer with a pH of 7–7.2 (0.1 M) was added, and the mixture was then subjected to micro-centrifugation at 12,000 rpm for 10 min at 4 °C. The supernatant was filtered with a nylon membrane. With this extract proteins, glutathione and ABTS (2,2´-azino-bis[3-ethylbenzthiazolin-6-sulfonic acid]) antioxidant capacity in hydrophilic compounds were determined.

The quantification of total protein (mg g^−1^ of dry weight (DW)) was performed using Bradford’s colorimetric technique [29]. In a microplate, 5 μL of the extract and 250 μL of Bradford reagent were placed in each well. The mixture was incubated for 10 min at room temperature (26 °C) and then read at a wavelength of 630 nm on a microplate reader (Allsheng, AMR-100 model, Hangzhou, China).

Lycopene and β-carotene (mg 100 g^−1^ DW) were determined according to Nagata and Yamashita [30]. For this, a sample (0.1 g) was mixed with 20 mL of hexane:acetone solution (3:2). An aliquot was taken from the supernatant and measured at absorbance values of 453, 505, 645, and 663 nm, as shown in Equations (1) and (2).

Lycopene = −0.0458 × Abs_663_ + 0.204 × Abs_645_ + 0.372 × Abs_505_ − 0.0806 × Abs_453_ (1)

β-carotene = 0.216 × Abs_663_ − 1.22 ×Abs_645_ − 0.304 × Abs_505_ + 0.452 × Abs_453_ (2)

Vitamin C (mg g^−1^ DW) was determined by the colorimetric method using 2,6 dichlorophenol, 1 g of fresh tissue, and 1 mL of 1% metaphosphoric acid and filtered with Whatman No. 1 filter paper, as described in Hung and Yeng [31].

Glutathione (mmol 100 g^−1^ DW) was determined using the method of Xue et al. [32] by means of a 5,5-dithio-bis-2 nitrobenzoic acid (DTNB) reaction. A mixture of 0.480 mL of the extract, 2.2 mL of sodium dibasic phosphate (Na_2_HPO_4_ at 0.32 M), and 0.32 mL of the DTNB dye (1 mM) was placed in a test tube. Then, the mixture was vortexed and read on a UV–Vis spectrophotometer (UNICO Spectrophotometer Model UV2150, Dayton, NJ, USA) at 412 nm using a quartz cell.

Flavonoids (mg 100 g^−1^ DW) were determined by the method of Arvouet-Grand et al. [33]. For the extraction, 100 mg of lyophilized tissue was placed in a test tube with 10 mL of reagent-grade methanol and then shaken for 30 s until the mixture was homogenized. The mixture was filtered using No. 1 Whatman paper. For the quantification, 2 mL of the extract and 2 mL of a methanolic solution of aluminum trichloride (AlCl_3_) 2% were added to the test tube and the mixture was left to rest for 20 min in the dark. The reading was then taken using a UV–Vis spectrophotometer (UNICO Spectrophotometer Model UV2150, Dayton, NJ, USA) at a wavelength of 415 nm using a quartz cell.

Phenols (mg g^−1^ DW) were determined with Folin–Ciocalteu reagent, as described in Cumplido-Nájera et al. [34]. The sample (0.2 g) was extracted with 1 mL of a water:acetone solution (1:1). The mixture was vortexed for 30 s. The tubes were centrifuged (UNICO Spectrophotometer Model UV2150, Dayton, NJ, USA) at 17,500× *g* for 10 min at 4 °C. In a test tube, 50 μL of the supernatant, 200 μL of the Folin–Ciocalteu reagent, 500 μL of 20% sodium carbonate (Na_2_CO_3_), and 5 mL of distilled water were added, and the mixture was then vortexed for 30 s. The samples were placed in a water bath at 45 °C for 30 min. Finally, the reading was taken at an absorbance of 750 nm using a plastic cell in a UV–Vis spectrophotometer (UNICO Spectrophotometer Model UV2150, Dayton, NJ, USA).

The antioxidant activity by ABTS was determined using the spectrophotometric method of Re et al. [35], which is based on the discoloration of the ABTS radical cation. This radical was obtained from the reaction of ABTS at 7 mM with potassium persulfate at 2.45 mM (1:1) in the dark at 26 °C for 16 h and then diluted with 20% ethanol to obtain an absorbance of 0.7 ± 0.01 at 750 nm. Afterwards, to determine antioxidant capacity in the hydrophilic compounds, phosphate buffer, 5 μL of extract, and 245 μL of the ABTS radical dilution (7 mM) were placed in a microplate, stirred for 5 s and then allowed to stand for 7 min in darkness. The absorbance was measured by a microplate reader (Allsheng, AMR-100 model, Hangzhou, China) at a wavelength of 750 nm. The blank was prepared with 250 μL of phosphate buffer (pH 7.0–7.2, 0.1 M). For the determination of the same in lipophilic compounds, extraction was carried out with a hexane:acetone solution. The results were expressed as vitamin C equivalents in mg per gram of dry weight (mg g^−1^ DW).

Hydrogen peroxide (µmol g^−1^ DW) levels were determined according to Velikova et al. [36] with slight modifications; 25 mg of lyophilized fruit tissue was weighed and placed in an Eppendorf tube, and 1 mL of cold 0.1% trichloroacetic acid was then added. The mixture was then centrifuged at 12,000× *g* for 15 min and 0.5 mL of the supernatant was taken; 0.5 mL of 10 mM potassium phosphate buffer (pH 7.0) and 1 mL of 1 M potassium iodide was added, and the reading was performed in a UV–Vis spectrophotometer (UNICO Spectrophotometer Model UV2150, Dayton, NJ, USA) at 390 nm. The H_2_O_2_ content was measured using a standard curve.

### 2.4. Arsenic Determination

To quantify the arsenic content in the organs of the plant (Root, stem, leaf and fruit), two methods were performed. The method for X-ray fluorescence with miniaturized tubes (ThermoScientific Niton XLt3, Boston, MA, USA) was used in all organs of the plant. The detection capacity of the equipment is 2 µg g^−1^. Analyses were performed in triplicate. The second method was used only in fruit—for this, acid digestion was performed with HNO_3_:H_2_O_2_. Subsequently, the samples were analyzed by inductively coupled plasma optical emission spectrophotometry ICP-OES (Varian Agilent 730-ES, Santa Clara, CA, USA). The arsenic detection capacity by the equipment is 1 µg L^−1^. 

### 2.5. Statistical Analysis

Six repetitions per treatment were analyzed, considering a fruit as an experimental unit. An analysis of variance (two-way ANOVA) was performed considering a completely randomized experimental design, and Fisher’s Least Significant Difference test was applied to compare the means (*p* ≤ 0.05). Additionally, Pearson’s correlation analysis was performed. The whole process was carried out using Infostat software (2018v) (https://www.infostat.com.ar).

## 3. Results

### 3.1. Arsenic Determination

**Figure 1:**
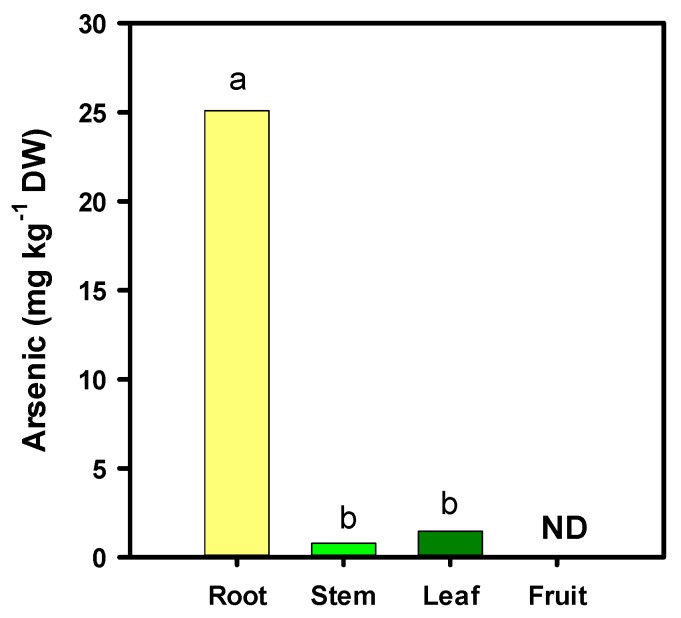
Arsenic concentration in different organs of the tomato plant. Different letters indicate significant differences according to Fisher’s Least Significant Difference test (*p* ≤ 0.05). ND: Not detected.

The X-ray fluorescence analysis showed the accumulation of arsenic only in the root, stem and leaves of tomato plants; however, in fruits, it was not detected by either of the two methods used (Figure 1). Therefore, it is possible that the arsenic has not been translocated to the fruits, or that it has been translocated in negligible amounts.

### 3.2. Fruit Quality

The quality of tomato fruits exposed to As and SiO_2_ NPs showed significant differences (Table 1). The firmness increased by 34.34% in the treatment with 1.6 mg L^−1^ of As and 250 mg L^−1^ of SiO_2_ NPs with respect to the control, although they were not statistically different. Regarding total soluble solids, there was a slight increase of 5.76% compared to the control with the treatment of 250 mg L^−1^ of SiO_2_ NPs, and also with 0.4 mg L^−1^ of As in the interaction with 1000 mg L^−1^ of SiO_2_ NPs. However, none of the treatments were statistically different. On the other hand, the pH decreased according to the exposure of As and SiO_2_ NPs alone and when they interacted, the control had the highest pH.

A tendency to increase the EC was observed at high doses of As, and in the interaction with the NPs, the treatment with 3.2 As and 250 SiO_2_ NPs mg L^−1^ resulted in an increase of 13.8%, while the low dose of As (0.2 mg L^−1^) showed a decrease (−4.07%) compared to the control, although they were not statistically different. Regarding the ORP, a decreasing tendency was observed as arsenic and SiO_2_ NPs were applied; the dose of 3.2 mg L^−1^ of As decreased the ORP by 72.25%.

### 3.3. Proteins

**Figure 2:**
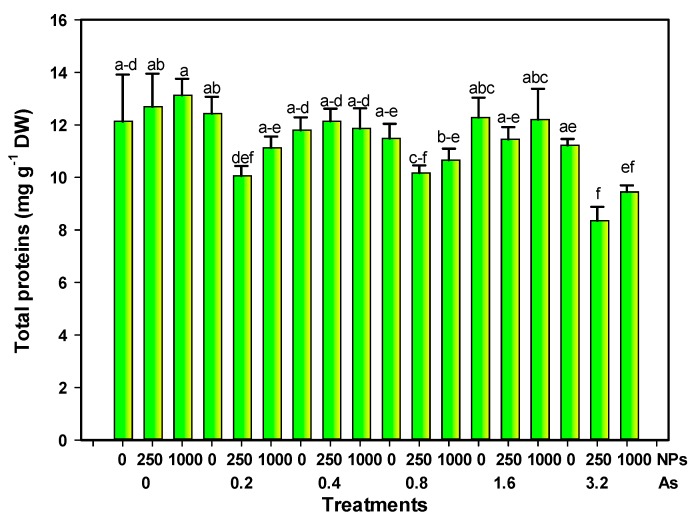
Total proteins in tomato fruits exposed to different doses of arsenic and nanoparticles. Different letters above the bars indicate significant differences according to Fisher’s Least Significant Difference test (*p* ≤ 0.05). NPs: Dose applied of SiO_2_ NPs (mg L^−1^); As: Dose applied of arsenic (mg L^−1^).

Total proteins decreased due to the interaction of the highest dose of As and SiO_2_ NPs, reaching up to 31.16% and 22.09% with 3.2–250 or 1000 mg L^−1^ of As and Si NPs, respectively, although the value also decreased by 16.15% with 0.8 and 250 mg L^−1^ of As and SiO_2_ NPs. The rest of the treatments were not statistically different from the control (Figure 2).

### 3.4. Antioxidant Compounds

**Figure 3:**
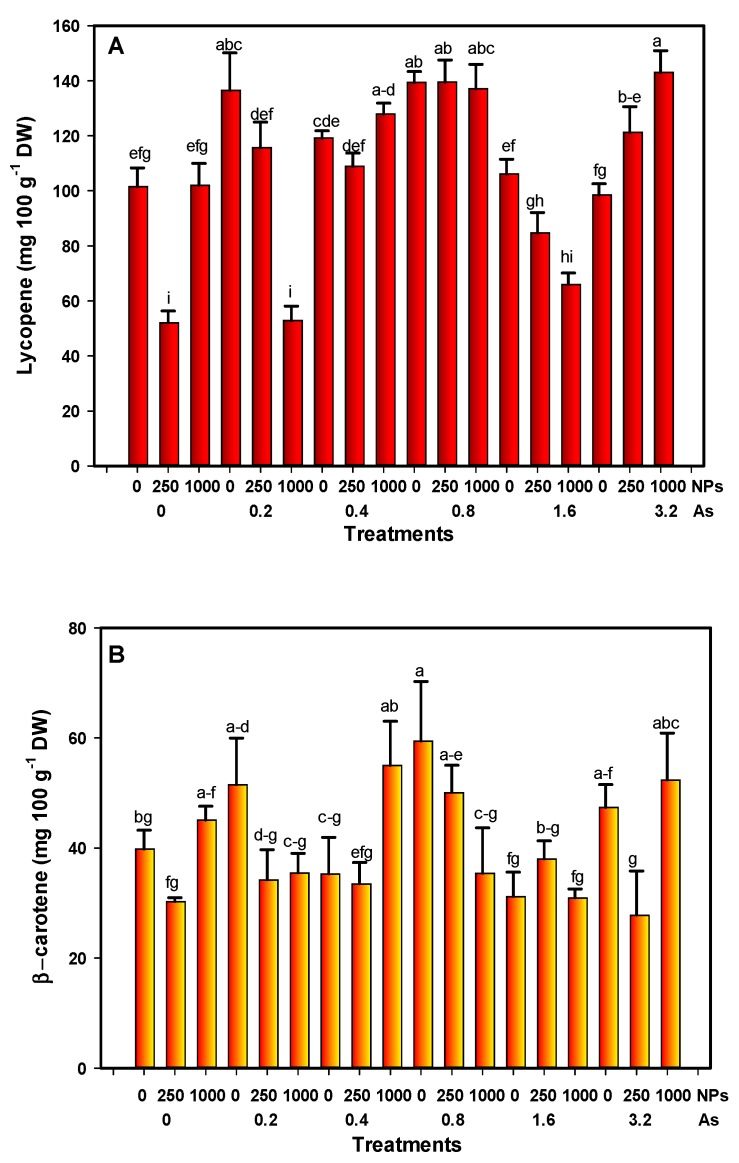
**(A**) Lycopene and (**B**) β-carotene content in fruits under stress conditions. Different letters above the bars indicate significant differences according to Fisher’s Least Significant Difference test (*p* ≤ 0.05). NPs: Dose applied of SiO_2_ NPs (mg L^−1^); As: Dose applied of arsenic (mg L^−1^).

The lycopene content in tomato fruits increased by 34% at the lowest dose of As (0.2 mg L^−1^). As–SiO_2_ NP interactions at 3.2–1000 mg L^−1^ increased lycopene by 40.82%. SiO_2_ NPs at 250 mg L^−1^ reduced the lycopene content by 51.30%, and 0.2 and 1000 mg L^−1^ of As–SiO_2_ NPs decreased it by 51.88% (Figure 3A).

Regarding the content of β-carotene, there was an increase with exposure to doses of 0.2, 0.8, and 3.2 mg L^−1^ of As without application of SiO_2_ NPs (29, 49, and 18%, respectively). An amount of 250 mg L^−1^ of SiO_2_ NPs decreased the content by 24%, although it was not statistically different from the control. The interaction between 3.2 and 250 mg L^−1^ As–SiO_2_ NPs decreased the β-carotene content by 40%. However, the dose of 1000 mg L^−1^ of SiO_2_ NPs without As increased the content of β-carotene by 13% (Figure 3B).

**Figure 4:**
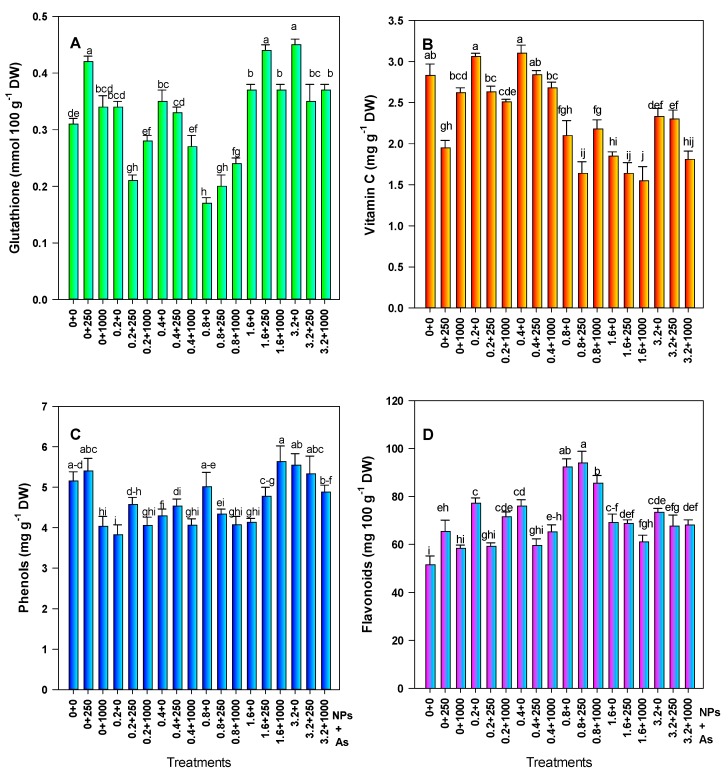
Non-enzymatic antioxidant compounds in tomato fruits. (**A**) Glutathione, (**B**) Vitamin C, (**C**) Phenols, and (**D**) Flavonoids. Different letters above the bars indicate significant differences according to Fisher’s Least Significant Difference test (*p* ≤ 0.05). NPs: Dose applied of SiO_2_ NPs (mg L^−1^); As: Dose applied of arsenic (mg L^−1^).

Glutathione increased with the application of 250 mg L^−1^ of SiO_2_ NPs (35.48%); however, this dose in combination with 0.2 and 0.8 mg L^−1^ of As decreased glutathione by 32.26% and 35.49%, respectively. The application of 0.8 mg L^−1^ of As decreased glutathione by 45.15%. When the plants were exposed to the two high doses of As and with the interaction with SiO_2_ NPs, there was a significant increase in glutathione with respect to the control. However, the greatest increase (45.16%) was in response to 3.2 mg L^−1^ of As without NPs, and 41.13% with 1.6–250 mg L^−1^ of As–SiO_2_ NPs (Figure 4A).

Regarding the vitamin C content, there was a 31.09% decrease when 250 mg L^−1^ of SiO_2_ NPs was applied. In addition, the three highest doses of As (0.8, 1.6, and 3.2 mg L^−1^) alone and in combination with the SiO_2_ NPs decreased the vitamin C content. The treatment of 0.8–250 mg L^−1^ of As–SiO_2_ NPs showed a 45.22% reduction in vitamin C, while treatments consisting of 1.6 and 3.2 mg L^−1^ of As in combination with 1000 mg L^−1^ of SiO_2_ NPs resulted in decreases of 42.02% and 36.04%, respectively. Although the doses of 0.2 and 0.4 mg L^−1^ of As alone showed a slight increase in this compound (8.12% and 9.54%, respectively), they were not statistically different from the control (Figure 4B).

Phenols decreased with low doses of As alone, as well as in combination with SiO_2_ NPs. The treatments with the greatest decrease in phenols were 0.2 mg L^−1^ of As (25.82%), as well as 0.2 and 0.4 mg L^−1^ in combination with 1000 mg L^−1^ of SiO_2_ NPs (21.35% and 21.16%, respectively). Regarding the effect of the SiO_2_ NPs, the dose of 250 mg L^−1^ showed a slight increase (4.85%); however, it was statistically the same as the control. In contrast, 1000 mg L^−1^ of SiO_2_ NPs decreased the phenol content by 21.74% (Figure 4C).

Flavonoids increased upon exposure to As and SiO_2_ NPs, although treatments with 1000 mg L^−1^ of SiO_2_ NPs alone, as well as 0.2 and 0.4 mg L^−1^ of As in combination with 250 mg L^−1^ SiO_2_ NPs were statistically the same as the control The greatest increase in flavonoids was with 0.8 mg L^−1^ of As alone, and in combination with 250 mg L^−1^ of SiO_2_ NPs—up to 79.26% and 82.42%, respectively (Figure 4D).

### 3.5. Antioxidant Capacity 

**Figure 5:**
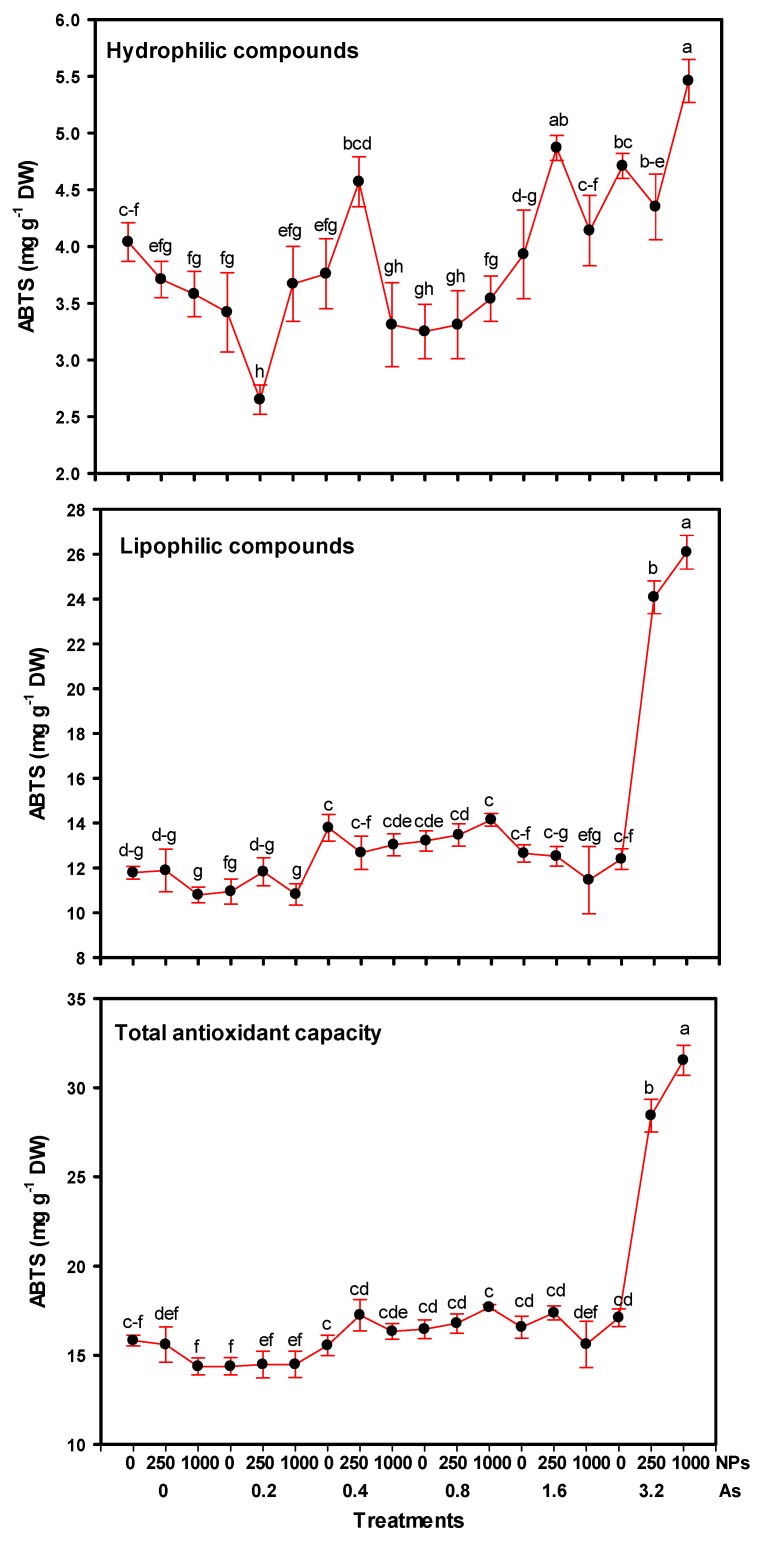
Antioxidant capacity of tomato fruits with applications of SiO_2_ NPs and stressed by arsenic. Different letters indicate significant differences according to Fisher’s Least Significant Difference test (*p* ≤ 0.05). NPs: Dose applied of SiO_2_ NPs (mg L^−1^); As: Dose applied of arsenic (mg L^−1^).

The antioxidant capacity determined in tomato fruits showed significant differences between treatments (Figure 5). With respect to hydrophilic compounds, 3.2–1000 and 1.6–250 mg L^−1^ of As–SiO_2_ NPs showed an increase of 35.14% and 20.54%, respectively. However, the antioxidant capacity decreased with 0.2–250 mg L^−1^ As–SiO_2_ NPs—by up to 34.4%. On the other hand, lipophilic compounds showed a similar trend to hydrophilic compounds; the highest dose of As (3.2 mg L^−1^) in combination with 250 and 1000 mg L^−1^ of SiO_2_ NPs being the treatments that presented respective increases of up to 104.24% and 121.20% in terms of antioxidant capacity.

Regarding the total antioxidant capacity, the highest dose of arsenic in combination with both doses of SiO_2_ NPs had the highest values, being 79.59% and 99.17% higher than the control, respectively.

### 3.6. Hydrogen Peroxide Content

**Figure 6:**
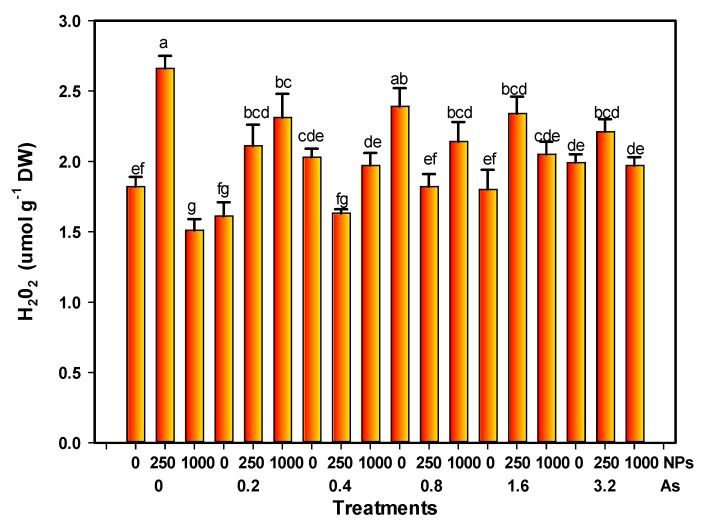
H_2_O_2_ content of tomato fruits with application of SiO_2_ NPs and stressed by arsenic. Different letters above the columns indicate significant differences according to Fisher’s Least Significant Difference test (*p* ≤ 0.05). NPs: Dose applied of SiO_2_ NPs (mg L^−1^); As: Dose applied of arsenic (mg L^−1^).

The H_2_O_2_ level increased by 46.15% with 250 mg L^−1^ of SiO_2_ NPs; however, it decreased by 17.05% with 1000 mg L^−1^ of SiO_2_ NPs compared with the control. On the other hand, 0.8 mg L^−1^ of arsenic alone increased the H_2_O_2_ level by 31.31%. Moreover, the interaction of 0.2 mg L^−1^ of As with 250 and 1000 mg L^−1^ of SiO_2_ NPs showed a slight increase of 15.93% and 26.92%, respectively (Figure 6).

Regarding the correlation between the different compounds evaluated, the results of the Pearson test showed high correlations between some variables, and only the SiO_2_ NPs did not show a correlation with any variable (Table 2). A positive correlation of As was observed with the antioxidant capacity of hydrophilic, lipophilic, total, and glutathione compounds (*r* = 0.53, 0.67, 0.71, and 0.41 (*p* ≤ 0.01), respectively); however, As had a negative correlation with proteins, vitamin C and ORP (*r* = −0.35 (*p* ≤ 0.05), −0.42, and −0.71 (*p* ≤ 0.01)). This suggests that arsenic is the treatment that most strongly influences the antioxidant content of the tomato fruits.

It was observed that the total antioxidant capacity had a highly positive correlation with the antioxidant capacity of lipophilic compounds (*r* = 0.99, *p* ≤ 0.01), while it was negatively correlated with proteins (*r* = −0.41, *p* ≤ 0.01) and ORP (*r* = −0.40, *p* ≤ 0.01).

Lycopene showed a positive correlation with the antioxidant capacity of lipophilic compounds (*r* = 0.33, *p* ≤ 0.05) and with the content of flavonoids (*r* = 0.39, *p* ≤ 0.01), while glutathione was positively correlated with the antioxidant capacity of hydrophilic compounds (*r* = 0.52, *p* ≤ 0.01). Likewise, it was observed that there was a negative correlation between lycopene and glutathione (*r* = −0.37, *p* ≤ 0.05).

Finally, the ORP had a negative correlation with the antioxidant capacity of hydrophilic compounds (*r* = −0.27, *p* ≤ 0.05), the antioxidant capacity of lipophilic compounds (*r* = −0.38, *p* ≤ 0.01), the total antioxidant capacity (*r* = −0.40, *p* ≤ 0.01), lycopene (*r* = −0.28, *p* ≤ 0.05), and flavonoids (*r* = −0.33, *p* ≤ 0.05); in contrast, it presented a positive correlation with proteins (*r* = 0.22, *p* ≤ 0.05) and vitamin C (*r* = 0.38, *p* ≤ 0.01).

## 4. Discussion

Considering the quality and safety of food products, the determination of toxic elements such as arsenic is important, since food contaminated with this element can cause health risks [37]. In the present study, arsenic was not detected in tomato fruits that were exposed to different concentrations of this element. This is because arsenic accumulates mainly in the root, while only a small portion is translocated to shoots with even smaller quantities to fruits [38]. Several studies have shown the presence of arsenic in fruits at very low concentrations [13,39,40], and so the risk for human consumption is very low.

The quality of fruits is indispensable for the market, particularly since fleshy fruits are perishable, and their quality is affected by different types of both abiotic and biotic stresses [41]. The observed effects are different depending on the variety, the phenological stage, the duration of stress, as well as the interaction with other environmental conditions [42]. The quality of the fruit is greatly modified during the ripening process in its different stages, affecting both the taste quality and the nutritional quality [43]. The initial decision to buy fruits and vegetables is usually made based on appearance and firmness [44]. In this sense, the firmness of the fruits increased consistently with the application of SiO_2_ NPs, coinciding with reports of other authors [45,46]. This is because silicon can accumulate in the epidermis, making it more rigid [47].

The application of SiO_2_ NPs decreased the pH of tomato fruits, which coincides with what was observed in jalapeño pepper with the application of Cu NPs + Cs–PVA [48]. The pH of the fruits directly influences the quality, in addition to the fact that consumers prefer less acidic fruits because they have a better flavor [49]. As for TSS and EC, in some studies, there was a decrease or increase in these parameters, depending on the type of treatment or the conditions to which the fruits were exposed [50,51].

The decrease in ORP observed in the As–SiO_2_ NPs interaction indicates a better fruit quality, since it can translate into a higher antioxidant potential [52], as observed in the correlations in this work. This trend has been reported by other authors who have worked with the application of NPs in tomato [28] and observed a decrease in ORP of 5.7% with the application of 250 mg L^−1^ of Cu NPs.

Proteins play an important role in regulating the development and quality of fruit [53]. Most of the regulatory proteins related to ripening in tomatoes have been functionally associated with the climacteric induction of ethylene biosynthesis [54]. Within all proteins, there are metallothioneins, which are proteins that respond to heavy metal stress [55]. Thiol groups in metallothioneins can act as powerful antioxidants, so they can play a role in protecting against oxidative damage [56]. However, when heavy metal concentrations are high, there is a higher generation of ROS, and therefore, greater oxidative stress [57], which can consequently decrease protein production. On the other hand, nanoparticles have been implicated in the increase in proteins in cucumber fruits [58]. However, they do not always have that effect, since it depends on the type of NPs and the dose used.

From the visual perspective, the quality of the fruit is directly related to the amount of carotenoids, mainly lycopene and β-carotene, that accumulate in ripe tomato fruits [59]. In addition, the consumption of carotenoids has beneficial effects on human health, since this can reduce the risk of certain forms of cancer, cardiovascular diseases, macular degeneration, among others [60]. In tomato fruits, ripening involves various morphological, physiological, biochemical, and molecular changes, including the degradation of chlorophyll as well as the synthesis and storage of carotenoids (mainly lycopene) during the transition from the chloroplast to the chromoplast [61]. Another important carotenoid is β-carotene, which is the main precursor of vitamin A; as opposed to β-carotene, lycopene has no pro-vitamin A activity but is a good antioxidant [62]. Lycopene is a powerful antioxidant and has a protective role against cancer [63]. In tomato fruits, the amount of lycopene can vary depending on the species, the stage of maturity, and the environmental conditions under which the fruit ripens [64]. The carotenoids in ripe fruit appear to play an important role in the detoxification of ROS, in particular to relieve the symptoms of stress induced by arsenic exposure [15], which is consistent with the results observed in this work.

Reduced glutathione (γ-glutamyl-cysteinyl glycine, GSH) participates in functional activities in many plants [65]. GSH has unique redox and nucleophilic properties and is involved in the cellular defense against the toxic actions of xenobiotics, oxiradicals, salinity, acidity, and metal cations [66]. Specifically under conditions of metal stress, GSH is a non-enzymatic antioxidant that plays a central role in homeostasis and acts as a precursor to a metal-binding peptide [67]. Therefore, a high concentration of GSH in cells acts as a buffer system against redox imbalance [68]. On the other hand, silicon can counteract the negative impact of oxidative stress by restricting the production of ROS caused by arsenic, in addition to improving the action of various antioxidant compounds and regulating the osmotic potential of the cell [69].

Vitamin C has a main function as an antioxidant and cofactor in redox reactions, in addition to being involved in the activation of epigenetic mechanisms that control cell differentiation [70]. However, vitamin C is highly unstable, and its levels may vary if subjected to stress factors [71]. Additionally, the antioxidants in the fruit can be affected by the intensity of light, temperature, and internal factors such as variety of cultivation, load, and position of the fruit [72]. For human health, sufficient absorption and distribution of vitamin C throughout the body are essential for many biochemical processes, including some that are vital to the growth and spread of tumors [73].

Phenols are antioxidant compounds that trigger the synthesis of a series of secondary metabolites from the shikimic acid pathway or through phenylpropanoids under conditions of abiotic stress [74]. The antioxidant capacity of phenolic compounds is also attributed to their ability to chelate metal ions involved in the production of free radicals [75]. Phenolic compounds can act as antioxidants, since hydroxyl groups donate hydrogen and can react with reactive oxygen species in a termination reaction, which breaks the cycle of generating new radicals [76].

It is known that silicon promotes the production of phenolic compounds that have an antioxidant or structural role. In addition, it can improve toxicity to heavy metals such as aluminum [77].

Flavonoids are among the most abundant phytochemicals in fruits and vegetables and have cancer cell anti-proliferative, antioxidant, anti-inflammatory, and estrogenic activities [78]. Flavonoids have anticancer activity in cellular and preclinical animal models, which makes them potential candidates in cancer prevention and treatment [79]. This is due to their antioxidants, which regulate the homeostasis of ROS [80].

Antioxidant capacity represents the ability to inhibit the oxidation process. It is a very desirable property of food since it plays an important role in various diseases [81]. There are different methods to evaluate the antioxidant capacity of a food; the two most commonly used tests are ABTS (2,2’-azino-bis-(3-ethylbenzothiazoline-6-sulfonic acid) and DPPH (2,2-diphenyl-1-picrylhydrazyl) [82]. The ABTS test can react with a wider range of antioxidant compounds [83]. Since the ABTS test is based on the generation of blue/green ABTS^•+^, which is applicable to both hydrophilic and lipophilic antioxidant systems, this method is better for the evaluation of the antioxidant capacity of highly pigmented and hydrophilic compounds compared to DPPH [84]. However, the antioxidant capacity of fruits to inhibit ABTS or DPPH also depends on the type of treatment to which they have been exposed [85].

ROS include free radicals, such as O^2−^ and OH, and non-radicals, such as H_2_O_2_ and ^1^O_2_, which induce oxidative damage in cells [86]. The increase in the H_2_O_2_ content is a typical plant response to arsenic exposure [87], so much so that when arsenic exposure increases, H_2_O_2_ levels also increase, being strongly time dependent [15]. One of the most prominent and earliest defense responses is the oxidative burst, generated by ROS, which includes H_2_O_2_ [88]. In addition to this, fruit ripening is an oxidative phenomenon that raises the level of ROS, a process that is genetically programmed [89]. Thus, the changes in the ROS of tomato fruits can be modified both by environmental and arsenic stress, as well as by the natural ripening process.

## 5. Conclusions

The results obtained in this research study highlight the importance of the stressful effect that arsenic can have on tomato fruit and how it modifies the antioxidant compounds. Low doses of As (0.2 mg L^−1^) in irrigation water induce a greater accumulation of antioxidant compounds; however, when exposed to high doses or when they interact with SiO_2_ NPs, there appears to be greater stress and oxidative damage that inhibit these compounds. On the other hand, 250 mg L^−1^ of SiO_2_ NPs increased glutathione. Flavonoids are the only group of antioxidants that are increased by As and SiO_2_ NPs. Total antioxidant capacity shows an increase with the highest dose of As in combination with SiO_2_ NPs. Our research shows that As, a highly toxic metalloid, and the SiO_2_ NPs, modified the processes of generating antioxidant compounds in ripe tomato fruit, usually with an increasing trend.

## Figures and Tables

**Table 1:** Quality of tomato fruits with applications of SiO_2_ nanoparticles (NPs) and stressed by arsenic.

As	SiO_2_ NPs	Firmness	TSS	pH	EC	ORP
(mg L^−1^)		(kg cm^−1^)	(°Brix)		(mS cm^−1^)	(mV)
0	0	4.60 ± 0.26 ^ab^	5.20 ± 0.28 ^ab^	4.75 ± 0.04 ^a^	3.98 ± 0.27 ^a–d^	54.83 ± 1.25 ^b^
	250	4.21 ± 0.85 ^b^	5.50 ± 0.34 ^a^	4.57 ± 0.02 ^bcd^	3.81 ± 0.15 ^bcd^	61.00 ± 1.79 ^a^
	1000	4.85 ± 0.46 ^ab^	5.33 ± 0.33 ^ab^	4.51 ± 0.02 ^def^	4.17 ± 0.16 ^abc^	51.50 ± 1.15 ^bc^
0.2	0	5.88 ± 0.56 ^ab^	5.25 ± 0.17 ^ab^	4.53 ± 0.02 ^b–f^	3.42 ± 0.47 ^d^	50.17 ± 2.09 ^c^
	250	4.35 ± 0.70 ^b^	5.17 ± 0.17 ^ab^	4.52 ± 0.03 ^b–f^	3.98 ± 0.24 ^a–d^	50.00 ± 1.69 ^cd^
	1000	5.88 ± 0.47 ^ab^	5.33 ± 0.21 ^ab^	4.51 ± 0.02 ^c–f^	4.30 ± 0.28 ^ab^	51.00 ± 0.89 ^bc^
0.4	0	4.97 ± 1.01 ^ab^	5.28 ± 0.16 ^ab^	4.58 ± 0.02 ^b^	3.51 ± 0.29 ^cd^	41.00 ± 1.32 ^e^
	250	5.45 ± 0.89 ^ab^	5.05 ± 0.03 ^ab^	4.52 ± 0.02 ^b–f^	4.19 ± 0.25 ^abc^	45.67 ± 0.92 ^d^
	1000	5.48 ± 0.24 ^ab^	5.50 ± 0.18 ^a^	4.51 ± 0.02 ^def^	3.87 ± 0.48 ^a–d^	40.17 ± 1.01 ^e^
0.8	0	5.27 ± 0.71 ^ab^	5.37 ± 0.20 ^ab^	4.47 ± 0.02 ^f^	4.22 ± 0.11 ^ab^	38.17 ± 1.96 ^efg^
	250	5.73 ± 0.57 ^ab^	5.23 ± 0.09 ^ab^	4.54 ± 0.02 ^b–e^	4.50 ± 0.14 ^ab^	38.83 ± 0.48 ^ef^
	1000	5.25 ± 0.54 ^ab^	5.17 ± 0.29 ^ab^	4.51 ± 0.01 ^c–f^	4.41 ± 0.18 ^ab^	39.83 ± 1.82 ^e^
1.6	0	5.31 ± 0.92 ^ab^	5.42 ± 0.20 ^ab^	4.52 ± 0.02 ^c–f^	4.25 ± 0.10 ^ab^	36.67 ± 1.87 ^efg^
	250	6.18 ± 0.39 ^a^	4.83 ± 0.17 ^b^	4.55 ± 0.02 ^b–e^	4.15 ± 0.13 ^abc^	37.17 ± 1.19 ^efg^
	1000	4.78 ± 0.56 ^ab^	5.33 ± 0.36 ^ab^	4.55 ± 0.01 ^b–e^	4.00 ± 0.36 ^a–d^	35.00 ± 2.35 ^fgh^
3.2	0	4.86 ± 0.57 ^ab^	5.42 ± 0.20 ^ab^	4.50 ± 0.04 ^ef^	4.14 ± 0.12 ^abc^	31.83 ± 2.15 ^h^
	250	5.73 ± 0.77 ^ab^	5.37 ± 0.20 ^ab^	4.54 ± 0.02 ^b–e^	4.53 ± 0.11 ^a^	36.83 ± 1.47 ^efg^
	1000	5.72 ± 0.46 ^ab^	5.03 ± 0.03 ^ab^	4.58 ± 0.02 ^bc^	4.15 ± 0.25 ^abc^	34.17 ± 1.72 ^gh^
CV (%)		30.05	10.30	1.29	14.92	9.04

As: arsenic; TSS: Total soluble solids; pH: Hydrogen potential; EC: Electric conductivity; ORP: oxidation reduction potential; CV: Coefficient of variation. Different letters within a column indicate significant differences according to Fisher’s Least Significant Difference test (*p* ≤ 0.05).

**Table 2:** Pearson correlations between As, SiO_2_ NPs, antioxidant capacity, H_2_O_2_, antioxidant compounds, proteins and ORP.

	ABTS H	ABTS L	TAC ABTS	H_2_O_2_	Lycopene	Β-Carotene	Proteins	Vitamin C	Glutathione	Phenols	Flavonoids	ORP
**As**	0.53 **	0.67 **	0.71 **	0.08 NS	0.14 NS	−0.02 NS	−0.35 *	−0.42 **	0.41 **	0.35 *	0.08 NS	−0.71 **
**SiO_2_ NPs**	0.04 NS	0.14 NS	0.14 NS	−0.04 NS	−0.12 NS	0.02 NS	−0.04 NS	−0.18 NS	−0.11 NS	−0.14NS	−0.13 NS	‒0.05
**ABTS H**		0.37 *	0.53 **	0.04 NS	−0.08 NS	0.01 NS	0.10 NS	−0.19 NS	0.52 **	0.33 *	−0.1 NS	−0.27 *
**ABTS L**			0.99 **	0.10 NS	0.33 *	−0.03 NS	−0.46 **	−0.22 *	0.07 NS	0.16 NS	0.04 NS	−0.38 **
**TAC ABTS**				0.10 NS	0.29 *	−0.02 NS	−0.41 **	−0.24 *	0.16 NS	0.20 *	0.02 NS	−0.40 **
**H_2_O_2_**					−0.22 *	−0.15 NS	−0.04 NS	−0.21 *	0.05 NS	0.31 *	0.19 NS	0.02 NS
**Lycopene**						0.19 NS	−0.19 NS	0.17 NS	−0.37 *	0.16 NS	0.39 **	−0.28 *
**β–carotene**							0.05 NS	−0.03 NS	−0.19 NS	0.01 NS	0.22 *	−0.10 NS
**Proteins**								0.11 NS	0.18 NS	−0.06 NS	−0.04 NS	0.22 *
**Vitamin C**									−0.09 NS	−0.22 *	0.17 NS	0.38 **
**GSH**										0.25 *	−0.28 *	0.11 NS
**Phenols**											−0.07 NS	−0.12 NS
**Flavonoids**												−0.33 *
		Significant Correlation				No significance						

*, ** Significant correlation at the *p* ≤ 0.05 and ≤ 0.01 levels, respectively. NS: no significance. ABTS H: Antioxidant capacity determined in hydrophilic compounds by 2,2´-azino-bis[3-ethylbenzthiazolin-6-sulfonic acid] radical, ABTS L: Antioxidant capacity determined in lipophilic compounds by 2,2´-azino-bis[3-ethylbenzthiazolin-6-sulfonic acid] radical, As: arsenic, SiO_2_ NPs: nanoparticles, H_2_O_2_: hydrogen peroxide, GSH: glutathione, and ORP: oxidation reduction potential.
